# Oral Administration of the Probiotic Strain *Escherichia coli* Nissle 1917 Reduces Susceptibility to Neuroinflammation and Repairs Experimental Autoimmune Encephalomyelitis-Induced Intestinal Barrier Dysfunction

**DOI:** 10.3389/fimmu.2017.01096

**Published:** 2017-09-14

**Authors:** Thomas Secher, Sahar Kassem, Mehdi Benamar, Isabelle Bernard, Michele Boury, Frederick Barreau, Eric Oswald, Abdelhadi Saoudi

**Affiliations:** ^1^IRSD, Université de Toulouse, INSERM, INRA, ENVT, UPS, Toulouse, France; ^2^Centre de Physiopathologie de Toulouse Purpan (CPTP), Université de Toulouse, UPS, INSERM, CNRS, Toulouse, France; ^3^CHU Toulouse, Hôpital Purpan, Service de Bactériologie-Hygiène, Toulouse, France

**Keywords:** *Escherichia coli* Nissle 1917, experimental autoimmune encephalomyelitis, probiotic, intestinal permeability, encephalitogenic T-cell, central nervous system

## Abstract

Multiple sclerosis (MS) is a chronic inflammatory disease of the central nervous system (CNS) with an increasing incidence in developed countries. Recent reports suggest that modulation of the gut microbiota might be one promising therapy for MS. Here, we investigated whether the probiotic *Escherichia coli* strain Nissle 1917 (ECN) could modulate the outcome of experimental autoimmune encephalomyelitis (EAE), a murine model of MS. We evidenced that daily oral treatment with ECN, but not with the archetypal K12 *E. coli* strain MG1655, reduced the severity of EAE induced by immunization with the MOG_35–55_ peptide. This beneficial effect was associated with a decreased secretion of inflammatory cytokines and an increased production of the anti-inflammatory cytokine IL-10 by autoreactive CD4 T cells, both in peripheral lymph nodes and CNS. Interestingly, ECN-treated mice exhibited increased numbers of MOG-specific CD4^+^ T cells in the periphery contrasting with severely reduced numbers in the CNS, suggesting that ECN might affect T cell migration from the periphery to the CNS through a modulation of their activation and/or differentiation. In addition, we demonstrated that EAE is associated with a profound defect in the intestinal barrier function and that treatment with ECN, but not with MG1655, repaired intestinal permeability dysfunction. Collectively, our data reveal that EAE induces a disruption of the intestinal homeostasis and that ECN protects from disease and restores the intestinal barrier function.

## Introduction

Multiple sclerosis (MS) is a chronic demyelinating inflammatory disease of the central nervous system (CNS) that predominantly affects young adults (1–3). The precise etiology of MS is still debated, but it is largely accepted that disease onset and progression result from a complex interplay between genetic and environmental factors (4–8). The role of the immune system in disease pathogenesis is indisputable, and a number of innate and adaptive immune cells are key for the development and progression of MS (9). MS is characterized by inflammation, demyelination, and neurodegeneration driven by autoreactive T cells possibly directed against myelin self-antigens (10, 11). CD4^+^ T cells that produce high levels of IFN-γ, IL-17, and GM-CSF are considered as important players in the immunopathogenesis of MS and its animal model, experimental autoimmune encephalomyelitis (EAE) (10, 12–14). In addition, MS patients exhibit defects in regulatory T cells (Tregs), known to suppress the activity of effector T cells (15).

In recent years, the incidence of autoimmune diseases in populations from developed countries has dramatically risen. One hypothesis is a modification of the gut microbiota, resulting from changes in diet combined with widespread introduction of antibiotics. Dysbiotic gut microbiota has been implicated in a wide range of immune-mediated diseases, including inflammatory bowel disease, MS, type 1 diabetes, and rheumatoid arthritis (16–19). In support of a role of a dysbiotic gut microbiota, the severity of EAE was reduced upon oral administration of antibiotics (20, 21). In a spontaneous relapsing–remitting mouse model of EAE, germ-free transgenic SJL/J mice were protected against disease, while the colonization of the gut by commensal microbiota restored susceptibility (22). Likewise, the mono-colonization of the gut of C57BL/6 mice with segmented-filamentous bacteria promoted Th17 accumulation in the spinal cord and restored EAE development (23). Together, these data suggest a role of indigenous gut microbiota in the pathogenesis of autoimmune diseases, thereby raising the possibility that modulation of gut microbiota could be used as a novel therapeutic approach. In this context, probiotics have been considered as potent modulators for the treatment of autoimmune diseases, due to the fact that they are generally recognized to confer beneficial health effects (24, 25). *Escherichia coli* strain Nissle 1917 (Mutaflor^®^, ECN) is a Gram-negative microorganism with probiotic properties that has been successfully used for the treatment of intestinal inflammation, especially in patients suffering from ulcerative colitis (26). In this study, we explored the therapeutic potential of ECN in CNS inflammatory disease, using the EAE model in mice. We show that ECN administration has beneficial effects on EAE and this beneficial effect is associated with changes in T cell compartmentalization, cytokines production by autoreactive CD4 T cells and intestinal permeability.

## Animals and Methods

### Animals

C57/BL6J male mice were obtained from Janvier Labs (Le Genest Saint-Isle, France) and housed under specific pathogen-free conditions with *ad libitum* access to food and water. All mice were housed under specific pathogen-free conditions at the INSERM animal facility (Zootechnie US-006), which is accredited by the French Ministry of Agriculture (accreditation number A-31 55508) to perform experiments on live mice. All experimental protocols were approved by the local ethics committee and are in compliance with the French and European regulations on care and protection of the Laboratory Animals (EC Directive 2010/63).

### Antibodies for Flow Cytometry and ELISA

The mAbs used for flow cytometry were as follows: RM4-5 (anti-mouse CD4), IM7 (anti-mouse CD44), H57-597 (anti-mouse TCRαβ), and FJK-165 (anti-mouse Foxp3). The fluorescent conjugated antibodies were purchased from e-Biosciences, BD Biosciences, and Biolegend. Antibodies used for ELISA were as follows: AN18 (anti-IFN-γ), purified anti-mouse IL-17A (BD Biosciences), purified anti-mouse GM-CSF (BD Biosciences), XMG1.2 (anti-IFN-γ biotin), biotin anti-mouse IL-17A (BD Biosciences), and biotin anti-mouse GM-CSF (BD Biosciences).

### Bacterial Preparation and Growth Conditions

The strains used in this study are the probiotic *E. coli* Nissle 1917 (ECN) and the archetypal K12 *E. coli* strain MG1655. Both strains were engineered to exhibit a mutation in the *rpsL* gene, which is known to confer resistance to streptomycin (27). Before oral administrations, ECN and MG1655 strains were grown for 6 h in LB broth supplemented with streptomycin (50 µg/ml) at 37°C with shaking. This culture was diluted 1:100 in LB broth without antibiotics and cultured overnight at 37°C with shaking. Bacterial pellets from this overnight culture were resuspended in sterile PBS to the concentration of 10^9^ cfu/ml. Bacteria were amplified from −80°C frozen stocks to ensure genetic stability.

### Induction of EAE and Clinical Investigation

The MOG_35–55_ (MEVGWYRSPFSRVVHLYRNGK) peptide was purchased from Polypeptide Laboratories (San Diego, CA, USA). 8- to 12-week-old mice were immunized subcutaneously with 50 µg of MOG_35–55_ peptide emulsified in complete Freund’s adjuvant (BD Difco, Franklin Lakes, NJ, USA) containing 500 µg of *Mycobacterium tuberculosis* (Strain H37, Difco). Mice were injected intravenously with 200 ng of pertussis toxin (List Biological Laboratories, Campbell, CA, USA) at days 0 and 2 post-immunization. Mice were daily treated with ECN, MG1655, or PBS starting 7 days before immunization (Figure S1 in Supplementary Material). Bacteria were administered every day by gavage at the dose of 10^8^ cfu/animal, prepared in 100 µl of PBS. Bacteria were freshly prepared every day as already described. Clinical scores were recorded daily as follow: 0, no signs of disease; 1, loss of tone in the tail; 2, hind limb paresis; 3, hind limb paralysis; 4, tetraplegia; and 5, moribund (28).

### Bacterial Load

Feces homogenates were prepared in 600 µl of isotonic saline solution using Precellys tissue homogenizer (Bertin Technologies). Ten-fold serial dilutions of homogenates were plated on MacConkey agar plates (Biovalley) supplemented or not with streptomycin. Plates were incubated at 37°C and 5% CO_2_ and cfu numbers were enumerated after 24 h. Colonies found growing on MacConkey’s agar plates without antibiotic were considered to be *enterobacteria*-like bacteria belonging to the family *Enterobacteriaceae*.

### Intestinal Permeability

*In vivo* intestinal permeability assessment was performed using FITC–dextran. Briefly, food and water were withdrawn for 3 h, and mice were gavaged with FITC–dextran (625 mg/kg body weight of FITC–dextran, MW: 4 kDa, FD4, Sigma-Aldrich). Serum was collected after retro-orbital puncture and fluorescence intensity was measured at 485/525 nm using an automatic Infinite M200 microplate reader (Tecan). FD4 concentrations were determined from standard curves generated by serial dilution of FD4. FD4 recovery upon time was calculated by linear regression of sample fluorescence, taking account of mice body weight (29).

*Ex vivo* intestinal permeability was performed in Ussing chamber. Biopsies from ileum or colon were placed in a chamber exposing 0.196 cm^2^ of tissue surface to 1.5 ml of circulating oxygenated Ringer solution at 37°C (30). Ileum and colon paracellular permeability were assessed by measuring the mucosal-to-serosal flux of 4 kDa FITC–dextran (Sigma-Aldrich) for 1 h (29). Results are expressed as flux of FITC–dextran per square centimeters of mucosa per hour.

### Real-time PCR

Total mRNA was extracted from ileum and colon mucosa scrapping by Trizol-Chloroform precipitation. mRNA was subsequently purified using Rneasy Plus kit (Qiagen) following the manufacturer’s instructions. For reverse transcription PCR, the iScript cDNA synthesis kit (Bio-Rad) was used. Real-time PCR was carried out using iQSYBR Green Supermix (Bio-Rad) for Reg3γ, Reg3β, ZO-1, Claudin-8, and IL-6. Primers are presented in Supplementary Table S1. Mouse glyceraldehyde 3-phosphate dehydrogenase served as endogenous control. PCR was done in duplicate and threshold cycle (Ct) values of the target genes were normalized to the endogenous control. Differential expression was calculated using the 2^−ΔΔCt^ methods and expressed as fold induction as compared to the expression in non-immunized animals (31).

### Isolation and Functional Characterization of Mononuclear Cells in CNS and Lymphoid Organs

Mice were anesthetized with ketamine and perfused with cold PBS. Brain and spinal cord were collected separately, homogenized and digested with collagenase D (2.5 mg/ml, Roche Diagnostics), Dnase I (10 µg/ml), and TLCK (1 µg/ml, Roche, Basel, Switzerland) for 30 min at 37°C. Cells were then washed, suspended in 37% Percoll, and layered on 70% Percoll. After a 20-min centrifugation at 2,000 rpm, the mononuclear cells were collected from the interface washed and resuspended in culture medium. For tetramer staining, cells from brain, spinal cord, and LNs were incubated for 2 h at room temperature with I-Ab MOG_38–49_ Tetramer-PE (NIH tetramer core facility) and then stained for surface markers before flow cytometry analysis. For functional analysis, 3 × 10^6^ total lymph node cells were stimulated with different concentrations of MOG_35–55_ for 72 h. Cytokines were then quantified in the supernatants.

### Cytokine Measurement

Enzyme immunoassays were used to measure cytokines in culture supernatants. 96-well plates were coated for 2 h at 37°C with anti-IFN-γ, anti-IL-17, or anti-GM-CSF in carbonate buffer 0.05 M pH 9.6. Culture supernatants or standards were incubated 2 h at 37°C. The plates were then incubated for 2 h with a secondary biotinylated antibody specific for each cytokine, followed by 20 min incubation with streptavidin–phosphatase alkaline at 37°C. Finally, plates were revealed by phosphatase alkaline substrate, and absorbance was measured at 450/540 nm. IL-10 and TNF were tested using CBA technology.

### Statistical Analyses

Statistical evaluation of differences between the experimental groups was determined by using two-way analysis of variance followed by a Bonferroni posttest for clinical monitoring and intestinal permeability assay; or with Mann–Whitney *U* test for the other assays. All tests were performed with GraphPad Prism 5.04 (GraphPad Software Inc., San Diego, CA, USA). All data are presented as mean ± SEM. A *p* value ≤0.05 was considered significant.

## Results

### ECN-Treated Mice Develop Less Severe EAE

**Figure 1:**
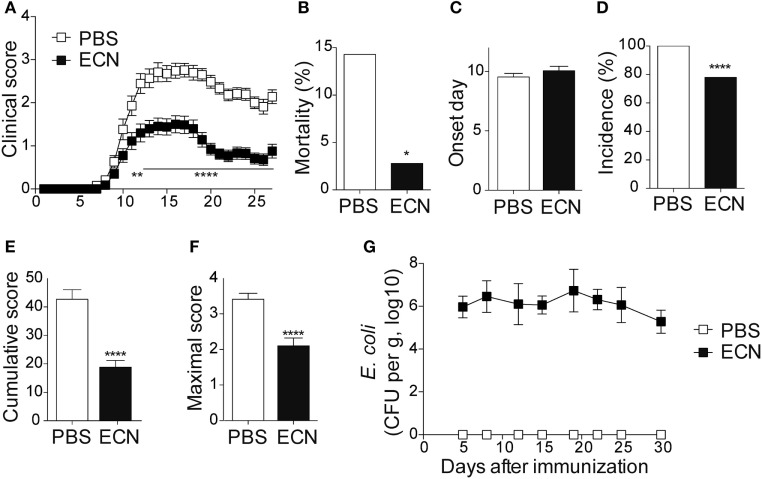
ECN-treated mice develop less severe experimental autoimmune encephalomyelitis (EAE). **(A)** Daily clinical score monitoring after MOG_35–55_ immunization. **(B)** Mortality during EAE progression. **(C)** Onset day assessment. **(D)** Disease incidence at day 30 after immunization. **(E)** Cumulative score was calculated as the sum of all EAE-clinical scores developed during the 30 days by each mouse per group. **(F)** Maximal scores were determined as the mean of maximal score reached by each mouse per group. **(G)** *Escherichia coli* enumeration in feces twice a week after immunization. Results are from three independent experiments and are depicted as means ± SEM (*n* = 40 mice per group), *p < 0.05; ****p < 0.001 comparing PBS-group (white bars) and ECN-group (black bars).

**Figure 2:**
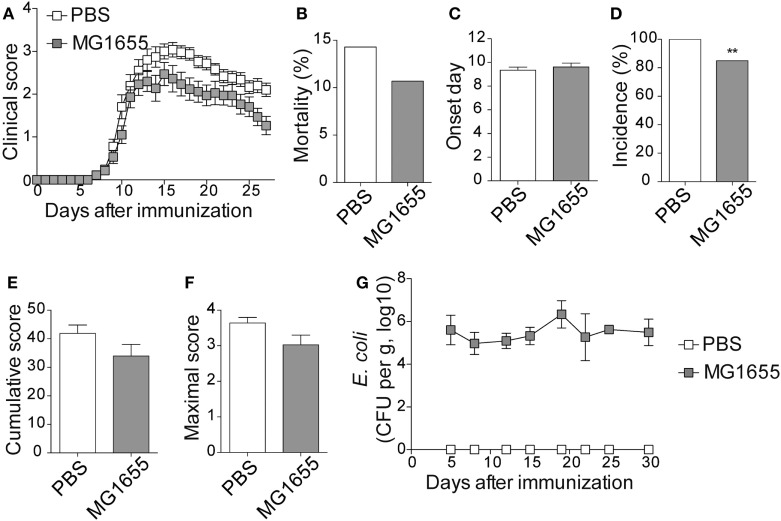
MG1655-treated mice are not protected from severe experimental autoimmune encephalomyelitis (EAE). **(A)** Daily clinical score monitoring after MOG_35–55_ immunization. **(B)** Mortality during EAE progression. **(C)** Onset day assessment. **(D)** Disease incidence at day 30 after immunization. **(E)** Cumulative score was calculated as the sum of all EAE-clinical scores developed during the 30 days by each mouse per group. **(F)** Maximal scores were determined as the mean of maximal score reached by each mouse per group. **(G)** *Escherichia coli* enumeration in feces twice a week after immunization. Data are from two independent experiments and are depicted as means ± SEM (*n* = 30 per group), **p < 0.01 comparing PBS-group (white bars) and MG1655-group (gray bars).

To study the impact of ECN treatment on the development of CNS inflammation, we compared the susceptibility of PBS- and ECN-treated mice to EAE according to the protocol shown in Figure S1 in Supplementary Material. When immunized by MOG_35–55_ peptide, PBS-treated animals developed a classical disease characterized by a progressive ascendant paralysis. Notably, when daily treated with ECN, mice developed a less severe disease, as illustrated by reduced clinical score (Figure 1A) and mortality (Figure 1B). The day of onset was similar between both groups (Figure 1C), while the incidence (Figure 1D) and the severity of disease progression were drastically reduced (Figures 1E,F) upon ECN treatment. Interestingly, mice treated with *E. coli* strain MG1655 developed similar disease progression, incidence and severity as PBS-treated animals (Figures 2A–F), suggesting that the protective effect observed with ECN is due to its intrinsic properties. The absence of probiotic effect with MG1655 is unlikely related to defective gut colonization, since *E. coli* intestinal loads were equivalent regardless of the type of strain (Figures 1G and 2G).

### Encephalitogenic MOG-Specific CD4^+^ T Cells Are Trapped in the Periphery in ECN-Treated Animals

**Figure 3:**
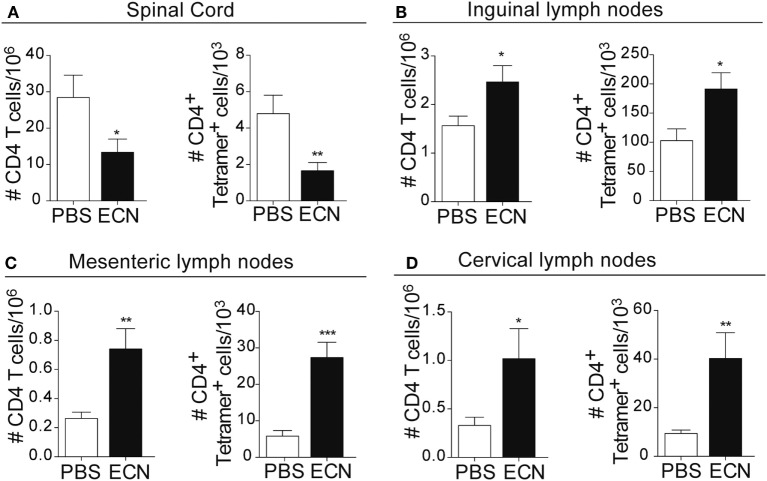
ECN-treated mice exhibit reduced migration of CD4^+^ T cell from the periphery to the central nervous system during the acute phase of experimental autoimmune encephalomyelitis. Absolute number of TCRαβ^+^CD4^+^ and TCRαβ^+^CD4^+^ MOG-specific Tetramer^+^ T cells isolated from the spinal cord **(A)**, draining lymph nodes **(B)**, mesenteric lymph nodes **(C)**, and cervical lymph nodes **(D)** 14 days after MOG_35–55_ immunization. Data are from three independent experiments and are depicted as means ± SEM (*n* = 20 per group for total CD4^+^ T cells and *n* = 5 per group for Tetramer^+^ T cells), *p < 0.05; **p < 0.01; ***p < 0.001 comparing PBS-groups (white bars) and ECN-groups (black bars).

**Figure 4:**
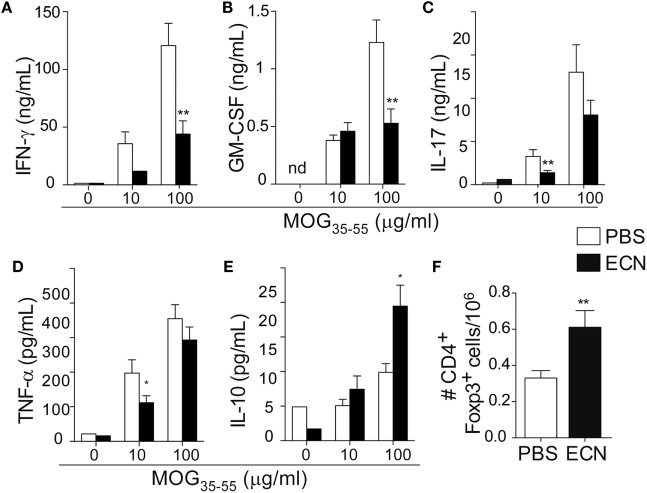
MOG-specific T cells present altered cytokine production in ECN-treated mice. Draining lymph node cells collected on day 14 after MOG_35–55_ immunization from PBS- or ECN-treated mice were stimulated *in vitro* with MOG_35–55_. Supernatants were collected after 72 h, and the secretion of IFN-γ **(A)**, GM-CSF **(B)**, IL-17 **(C)**, TNF **(D)**, and IL-10 **(E)** was determined by ELISA or CBA. **(F)** Absolute number of TCRαβ^+^CD4^+^CD25^+^FoxP3^+^ regulatory T cells isolated from draining lymph nodes. Results are from three independent experiments and are depicted as means ± SEM (*n* = 12–14 per group for cytokines and *n* = 10–15 for FoxP3^+^ T cells). *p < 0.05; **p < 0.01 comparing PBS-group (white bars) and ECN-group (black bars).

Peripheral activation and subsequent migration of autoreactive CD4^+^ T cells into the CNS are critical steps in the pathogenesis of EAE and MS (32, 33). 14 days after EAE induction, leukocytes were isolated from the spinal cord or peripheral lymphoid organs and were analyzed. Interestingly, the total numbers of CD4^+^ and MOG-specific CD4^+^ T cells were significantly decreased in the spinal cord (Figure 3A) of ECN-treated animals. In contrast, the numbers of these cells increased in inguinal (draining) (Figure 3B), mesenteric (Figure 3C), and cervical (Figure 3D) lymph nodes in ECN-treated animals. We further checked whether oral treatment with ECN could modulate the effector function of MOG-specific CD4^+^ T cells. Draining lymph node T cells collected on day 14 (Figure 4) or day 30 (Figure S2 in Supplementary Material) after immunization were stimulated with MOG_35–55_ for 72 h and analyzed for the production of various cytokines. Treatment with ECN significantly reduced the production of IFN-γ, GM-CSF, IL-17, and TNF-α (Figures 4A–D; Figure S2 in Supplementary Material). This effect was not observed when T cells originated from MG1655-treated mice (Figure S2 in Supplementary Material). Intriguingly, treatment with ECN significantly increased the production of IL-10 by MOG-specific CD4^+^ T cells (Figure 4E), which is a hallmark of Tregs (34, 35). Moreover, draining lymph nodes from ECN-treated mice exhibited a significantly higher number of CD4^+^Foxp3^+^ cells (Figure 4F). Taken together, these data demonstrate that ECN protection of the host from CNS inflammation is associated to dampened inflammatory cytokine production and reduced migration of MOG-specific T cells from the periphery to the CNS.

### EAE-Mediated Perturbation of the Intestinal Barrier Function Is Prevented by ECN Treatment

**Figure 5:**
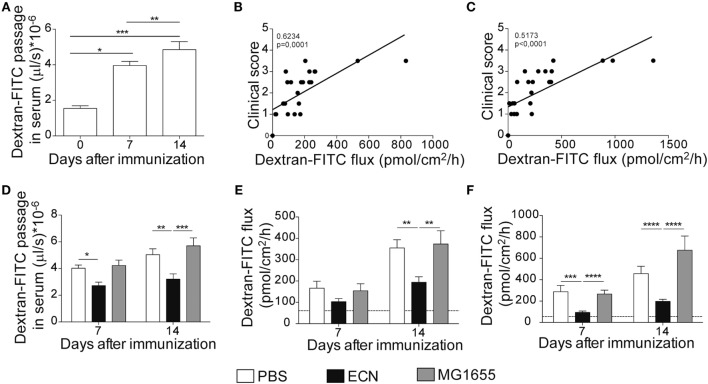
Experimental autoimmune encephalomyelitis (EAE) severity is associated with increased intestinal permeability prevented by ECN treatment. FD4 recovery in the serum at day 0, 7, and 14 after MOG_35–55_ immunization of mice daily treated with PBS **(A)**. Data are from three independent experiments and are expressed as means ± SEM (*n* = 20 per group), *p < 0.05; **p < 0.01; ***p < 0.001. Correlation between EAE-clinical scores and intestinal permeability of the colon **(B)** and the ileum **(C)** at day 14 after MOG_35–55_ immunization of mice daily treated with PBS. **(D)** FD4 recovery in the serum at day 7 and 14 after MOG_35–55_ immunization of mice treated daily with PBS (white), ECN (black), or MG1655 (gray). Colon **(E)** and ileum **(F)** biopsies were mounted in Ussing chambers at day 7 and 14 after MOG_35–55_ immunization, and paracellular permeability was monitored. Data are from two independent experiments and are expressed as means ± SEM (*n* = 8 per group), *p < 0.05; **p < 0.01; ***p < 0.001; ****p < 0.0001.

It has been recently reported that intestinal permeability is increased during the course of EAE (36). In agreement with these studies, mice exhibited a robust and time-dependent enhanced intestinal permeability when immunized with MOG_35–55_, as assessed *in vivo* by the luminal to blood passage of fluorescent marker [FITC–dextran 4 kDa (FD4)]. This was observed before the clinical onset, i.e., 7 days after immunization and at the peak of the disease, 14 days after immunization (Figure 5A). Interestingly, we observed a significant correlation between colon and ileum permeability and EAE-clinical score 14 days after immunization (Figures 5B,C), suggesting that intestinal barrier dysfunction is an important parameter accounting for the severity of autoimmune inflammation.

**Figure 6:**
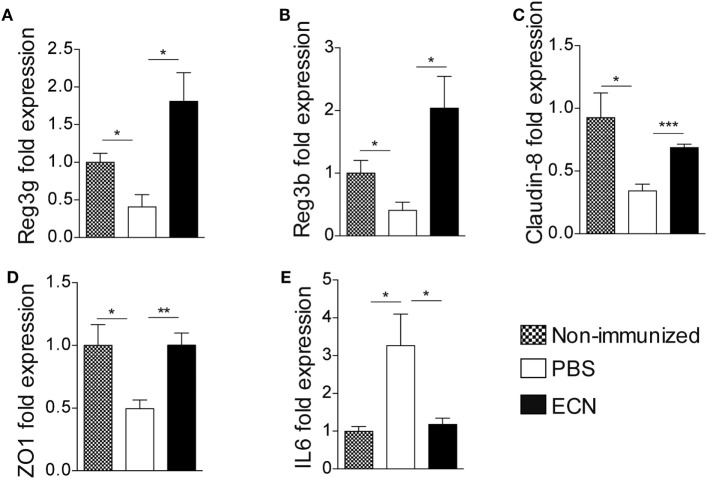
ECN treatment protects from experimental autoimmune encephalomyelitis-mediated alteration of the intestinal barrier function. Real-time PCR analysis of Reg3γ **(A)**, Reg3β **(B)**, Claudin-8 **(C)**, ZO-1 **(D)**, and IL-6 **(E)** in the ileum mucosa 14 after MOG_35–55_ immunization of mice daily treated with PBS, ECN or left unimmunized. Data are from two independent experiments and are expressed as means ± SEM (*n* = 8 per group), *p < 0.05; **p < 0.01; ***p < 0.001; comparing PBS-groups (white bars), ECN-groups (black bars), and unimmunized group (dashed bars).

The effectiveness of ECN as a probiotic organism has been correlated to its ability to strengthen the intestinal barrier function (37). To investigate the impact of ECN treatment on intestinal barrier function during EAE progression, we measured the intestinal permeability of mice treated daily with ECN, 7 and 14 days after MOG_35–55_ immunization. The oral treatment with ECN significantly reduced the FD4 passage from the intestinal lumen to the blood compartment, demonstrating that ECN is able to preserve the intestinal barrier function during EAE (Figure 5D). In contrast, mice treated with MG1655 developed similar intestinal barrier function impairment as PBS-treated animals (Figure 5D). To gain further insight into EAE-induced intestinal barrier dysfunction, colon and ileum biopsies were mounted in Ussing chambers and analyzed for the paracellular flux of FD4. Seven and fourteen days after immunization, PBS-treated mice exhibited an increase of both colon and ileum permeability (Figures 5E,F). Such an increase in colon and ileum intestinal permeability was not modulated by MG1655 treatment, while they were significantly reduced by oral treatment with ECN (Figures 5E,F). To investigate the underlying mechanisms, we first analyzed several components of the intestinal barrier function. Fourteen days after MOG_35–55_ immunization, we observed a downregulation of mRNA coding for the antimicrobial peptides Reg3γ (Figure 6A) and Reg3β (Figure 6B), as well as for the tight-junction proteins Claudin-8 (Figure 6C) and ZO-1 (Figure 6D), whereas expression of the prototypal pro-inflammatory cytokine IL-6 was increased (Figure 6E). These alterations of the intestinal barrier function were reduced upon ECN treatment as compared to PBS or MG1655-treated animals (Figure 6; Figure S3 in Supplementary Material).

## Discussion

Intestinal immune homeostasis has recently emerged as a critical cofactor in autoimmune processes (38). The intimate equilibrium between intestinal microflora and gut barrier function is often modified during chronic inflammatory and autoimmune diseases, resulting in efforts to restore the microbial composition by using probiotics. In this study, we investigated the efficacy and the mechanism supporting the effect of *E. coli* Nissle 1917 on neuroinflammation, using the EAE model. We have shown here that a prophylactic daily treatment with ECN drastically reduced the development, progression, and severity of CNS inflammation and this beneficial effect is associated with changes in T cell compartmentalization, cytokines production, and intestinal permeability.

Probiotics are defined as live bacteria that can give a health benefit to the host when administered in adequate amounts (39). Multiple mechanisms have been described to support their beneficial effects, including inhibition of the adhesion and invasion of epithelial cells by pathogens, modulation of intestinal inflammatory responses and improvement of the intestinal barrier (40). ECN is one of the most investigated probiotic bacteria (41). Numerous studies have demonstrated its beneficial effects in the management of intestinal inflammatory disorders, especially ulcerative colitis (41). Despite a rich literature, the exact mechanism underlying ECN efficiency is poorly understood, and preclinical studies addressing its efficacy in CNS disease are lacking. Here, we show for the first time that ECN treatment has beneficial effects on CNS inflammation by reducing disease severity and mortality. In contrast, mice treated with the archetypal K12 *E. coli* strain MG1655 were as sensitive to EAE as untreated mice suggesting that the genetic background of the strain is of particular importance and determines its ability to act as a probiotic.

One of the best-characterized feature that is likely to contribute to the effectiveness of ECN as a probiotic organism is its ability to strengthen the gut barrier function (42). While several autoimmune pathologies have been associated with perturbation of intestinal barrier function (38), the correlation between a “leaky gut” syndrome and the development of MS is yet unknown. It has been suggested that patients with relapsing–remitting (RRMS) or secondary-progressive MS exhibit increased intestinal permeability (38, 43), whereas RRMS patients under remission show a restored intestinal permeability almost comparable to controls (38). Recently, Nouri et al. revealed that intestinal permeability was altered during EAE (36). In agreement with this study, we observed that EAE provoked increased paracellular permeability at ileal and colonic locations. Of note, recent reports showed that patients suffering from IBD, another “leaky gut”-related immune-mediated syndrome, for which ECN treatment is protective, were at higher risk for the development of MS (44). We also provided evidence that the increase of both small and large intestine permeability was associated with altered mRNA expression of intestinal proteins that are critical for the strengthening of the physical and biochemical intestinal barriers and for the homeostasis of the gut microbiota, including tight-junction proteins (ZO-1 and Claudin-8) and antimicrobial peptides (Reg3β and Reg3γ) (45, 46). In addition, the expression of IL-6 was significantly increased in the gut mucosa upon EAE immunization. This pro-inflammatory cytokine is markedly increased in various intestinal inflammatory diseases and has been associated with increased tight-junction permeability (47). Of note, we demonstrated that the sealing action of ECN improves intestinal barrier function through the upregulation of tight-junction proteins and antimicrobial peptides. Interestingly, we observed a correlation between the perturbation of the intestinal barrier function and the severity of the neurological syndromes, further supporting the fact that ECN-dependent improvement of intestinal permeability may modulate or perpetuate CNS autoimmunity.

The alterations of the intestinal barrier have a major consequence: the increased transmucosal passage of putative noxious or immunogenic antigens. The intestinal mucosa is a unique environment for dialog between microbiota-derived antigens and the mucosal immune system. This cross talk is now known to affect immunological homeostasis and tolerance within the gut (48), but also in systemic compartment by modulating T cell priming and activation (49). Indeed, recent studies have demonstrated that RRMS patients exhibited alterations in their gut microbiota composition (19, 50) and that these dysbiotic microbial populations profoundly altered gene expression of monocytes and T cells, which are involved both in the initiation phase of immunity and in the activation of the adaptive immune response (19). In this study, we showed that ECN treatment during EAE affects T cell homeostasis. In contrast to the CNS, there was an increase in the total number of MOG-specific CD4^+^ T cells in the inguinal, cervical and to larger extent mesenteric lymph nodes of mice treated with ECN. These CD4^+^ T cells presented an altered function as evidenced by their decreased production of IFN-γ, IL-17, GM-CSF, and TNFα, while the secretion of IL-10 was increased and this was associated with an enriched population of CD4^+^CD25^+^FoxP3^+^ Tregs. Thus, our results suggest that the mechanism underlying the resistance to EAE of ECN-treated animals could results from a defective differentiation of autoreactive CD4^+^ T cell in the periphery, which is known to account for their egress from the periphery to the CNS (51, 52). Our study is also consistent with previous ones showing that colonization by ECN lead to a modification of the cytokine repertoire, with an increased production of IL-10 by monocytes or activated lymphocytes (53, 54). This anti-inflammatory feature is shared by other probiotic strains that often lead to increased IL-10 production (55, 56). The upregulation of IL-10 production in ECN-treated mice may be one of the mechanisms preventing inflammation and autoimmunity. In this regard, EAE recovery is associated with increased production of IL-10, which is known to suppress EAE (57, 58).

The molecular rationale behind the immunomodulatory effect of ECN in EAE has not yet been demonstrated and is under further investigation by our team. The beneficial effect of ECN in CNS inflammation could be explained by the improvement of the intestinal barrier function and the resulting prevention of a continuous stimulation of the host innate immune system by the gut components. In this regard, it has been shown that microbiota-associated molecular patterns can activate the host innate immune system *via* pattern-recognition receptors, such as toll-like receptors (TLRs) and nucleotide-binding domain and leucine-rich repeat containing receptors [NOD-like receptors (NLRs)] present in intestinal epithelial and myeloid cells (59). Thus, the activation of TLRs and NLRs could be implicated in the mechanisms by which gut microbiota trigger autoimmune diseases. It is also possible that ECN may reduce microbial imbalance in the EAE gut through inter or intraspecies competition, for which ECN genome encodes specialized molecules (60).

In conclusion, our findings show that alteration of the intestinal barrier function is associated with EAE progression and severity. Our results also suggest that the improvement of the intestinal permeability by probiotic treatment may be a useful way to control neuroinflammation and CNS pathogenesis. These results pave the way to consider ECN as valuable and innovative prophylactic or diet supplements to modulate or reverse the initiation and progression of CNS inflammatory diseases or other immunological disorders associated with alterations in the epithelial barrier of the gut, the so-called leaky gut.

## Ethics Statement

This study was carried out in accordance with the recommendations of the French and European regulations on care and protection of the Laboratory Animals (EC Directive 2010/63). The protocol was approved by the local ethics committee.

## Author Contributions

TS and AS conceived of the project; SK, TS, FB, EO, and AS designed and performed the research and analyzed and interpreted the data; MBenamar, IB, and MBoury facilitated the research; TS, SK, EO, and AS prepared the figures and wrote the manuscript.

## Conflict of Interest Statement

The authors declare that the research was conducted in the absence of any commercial or financial relationships that could be construed as a potential conflict of interest.

## Abbreviations

CFA, complete Freund’s adjuvant; CNS, central nervous system; EAE, experimental autoimmune encephalomyelitis; ECN, *Escherichia coli* Nissle 1917; FD4, fluorescein isothiocyanate-dextran 4 kDa; MS, multiple sclerosis; NLR, NOD-like receptor; Treg, regulatory T cell; TLR, toll-like receptor.
