# Supplementary figures and images for: Oral Administration of the Probiotic Strain Escherichia coli Nissle 1917 Reduces Susceptibility to Neuroinflammation and Repairs Experimental Autoimmune Encephalomyelitis-Induced Intestinal Barrier Dysfunction
Source: Front Immunol. 2017 Sep 14;8:1096. doi: 10.3389/fimmu.2017.01096 (PMC5603654; doi:10.3389/fimmu.2017.01096)

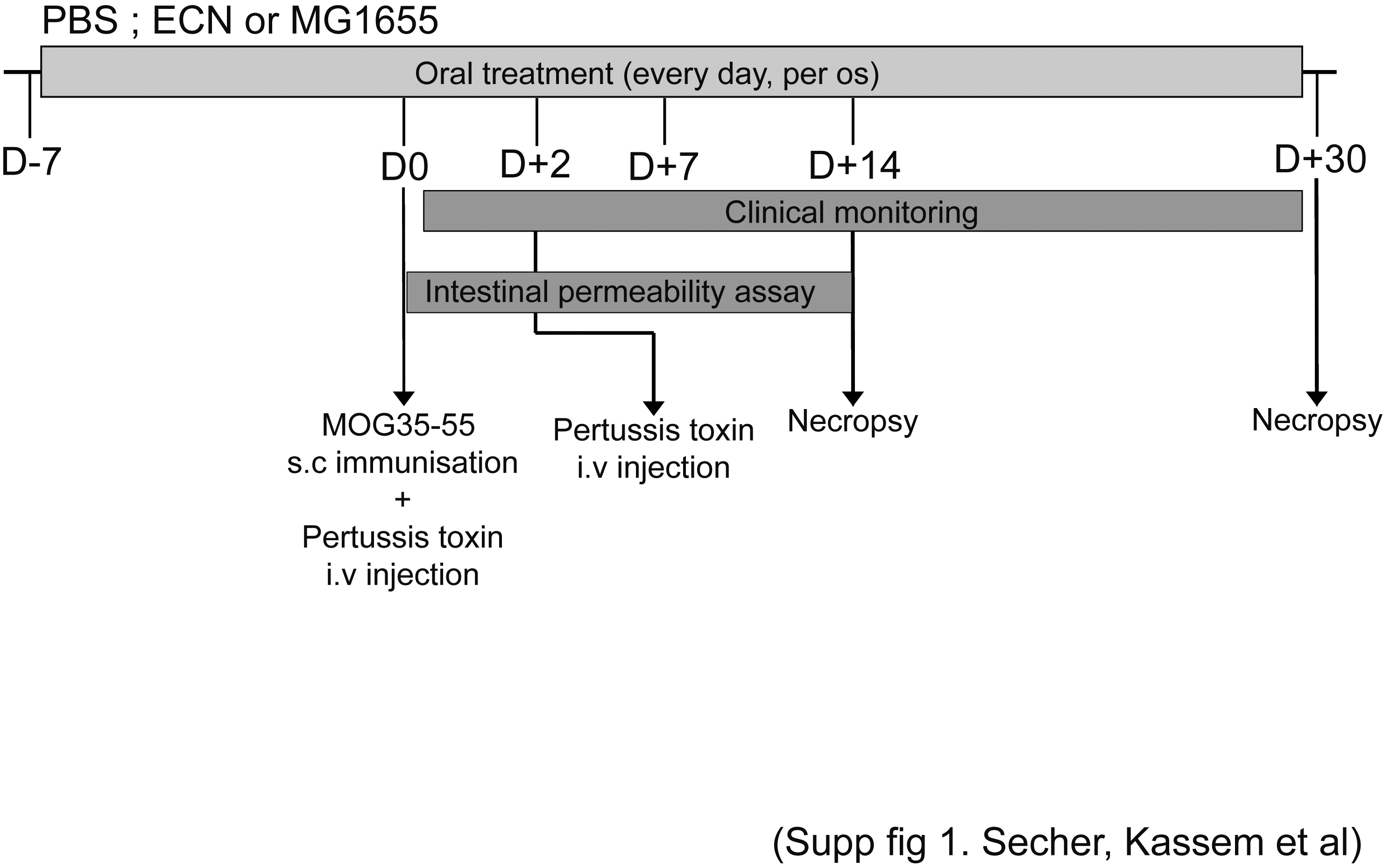

Supplement: Supplementary file 1 [file Data_Sheet_1.DOCX]
