# Supplementary material for: Oral Administration of the Probiotic Strain Escherichia coli Nissle 1917 Reduces Susceptibility to Neuroinflammation and Repairs Experimental Autoimmune Encephalomyelitis-Induced Intestinal Barrier Dysfunction
Source: Front Immunol. 2017 Sep 14;8:1096. doi: 10.3389/fimmu.2017.01096 (PMC5603654; doi:10.3389/fimmu.2017.01096)
Supplement: Supplementary file 2 [file Data_Sheet_2.DOCX]

**
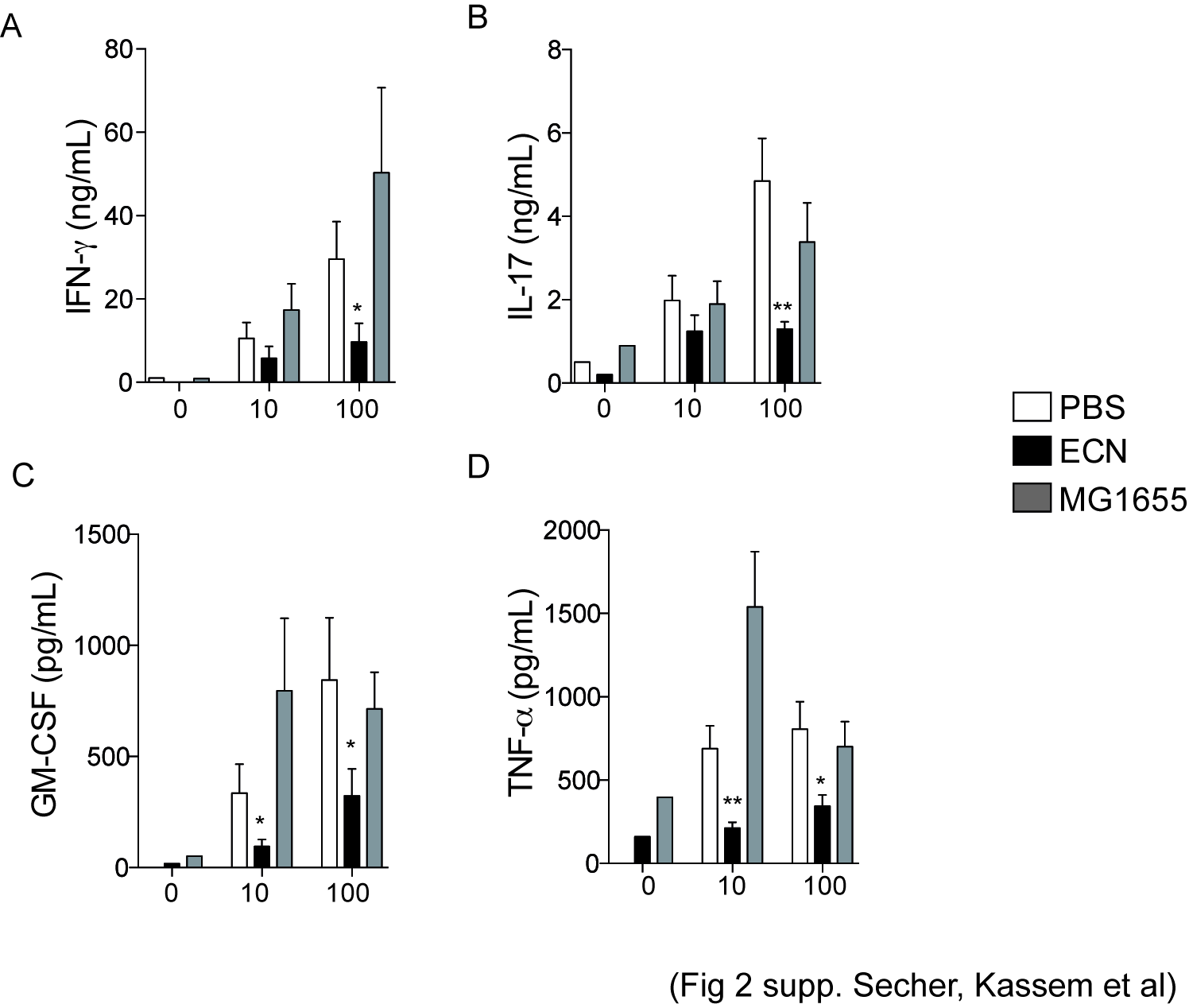
**

**Supplementary figure 2: MOG-specific T cells present altered cytokine production in ECN- but not in MG1655- treated mice**

Draining lymph node cells collected on day 30 after MOG_35-55_ immunization from PBS- ; ECN- or MG1655- treated mice were stimulated *in vitro* with MOG_35-55_. Supernatants were collected after 72 h and the secretion of IFN-γ (A), IL-17 (B), GM-CSF (C) and TNF (D) was determined by ELISA. Results are depicted as means ± s.e.m. (n=8-10 per group). *p<0.05; **p<0.01 comparing PBS-group (white bars) and ECN-group (black bars) and MG1655-group (grey bars).
