# Supplementary material for: Oral Administration of the Probiotic Strain Escherichia coli Nissle 1917 Reduces Susceptibility to Neuroinflammation and Repairs Experimental Autoimmune Encephalomyelitis-Induced Intestinal Barrier Dysfunction
Source: Front Immunol. 2017 Sep 14;8:1096. doi: 10.3389/fimmu.2017.01096 (PMC5603654; doi:10.3389/fimmu.2017.01096)
Supplement: Supplementary file 3 [file Data_Sheet_3.DOCX]

**
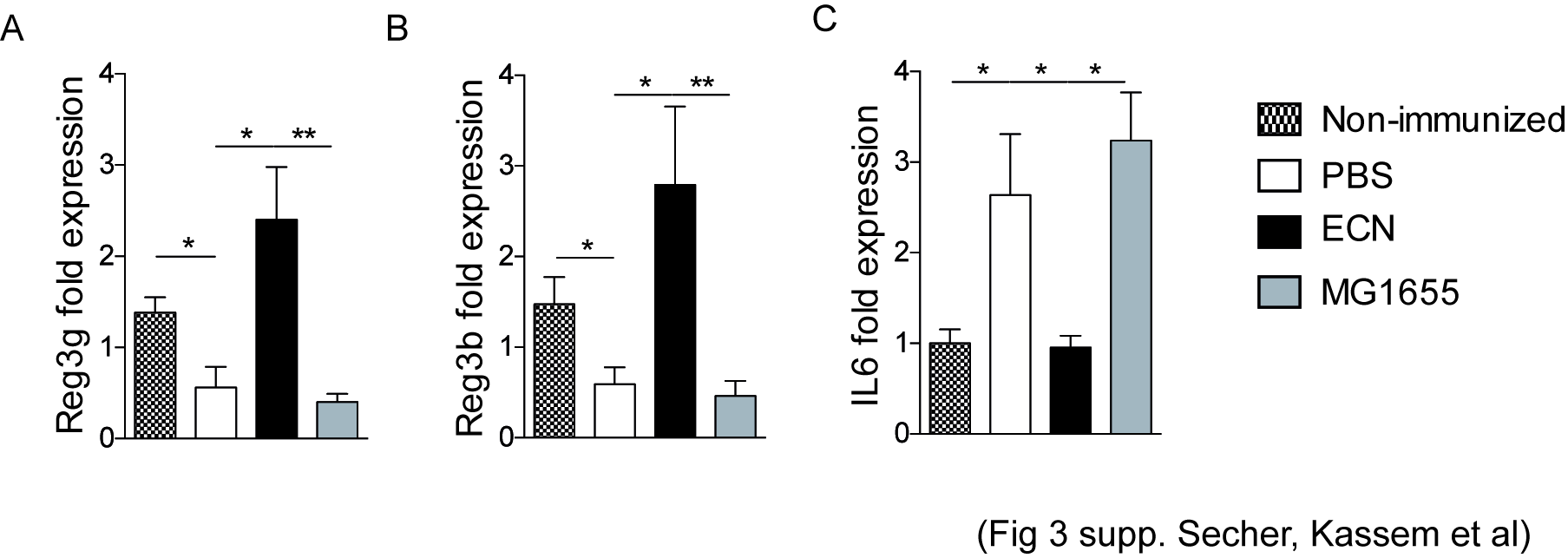
**

**Supplementary figure 3: ECN, but not MG1655, treatments protect from EAE-mediated alteration of the intestinal barrier function**

Real-time PCR analysis of Reg3γ (A), Reg3β (B), and IL-6 (C) in the ileum mucosa 14 after MOG_35-55_ immunization of mice daily treated with PBS, ECN, MG1655 or left unimmunized. Data are from two independent experiments and are expressed as means ± s.e.m. (n=4-8) per group), *p<0.05; **p<0.01; comparing PBS-groups (white bars), ECN-groups (black bars), MG1655-groups (grey bars) and unimmunized group (dashed bars).
