# Supplementary material for: Oral Administration of the Probiotic Strain Escherichia coli Nissle 1917 Reduces Susceptibility to Neuroinflammation and Repairs Experimental Autoimmune Encephalomyelitis-Induced Intestinal Barrier Dysfunction
Source: Front Immunol. 2017 Sep 14;8:1096. doi: 10.3389/fimmu.2017.01096 (PMC5603654; doi:10.3389/fimmu.2017.01096)
Supplement: Supplementary file 4 [file Table_1.DOCX]

| \|  \| \| --- \| |  |  |  |
| --- | --- | --- | --- | --- |
| **Supplementary Table 1** | | |  |
| Gene name |  | Primer sequence (5'-3') |  |
| \|  \| \| --- \| |  |  |  |
| Reg3γ |  | CCTCAGGACATCTTGTGTCTGTGCTC |  |
|  |  | TCCACCTCTGTTGGGTTCATAGCC |  |
| Reg3β |  | CAGACCTGGTTTGATGCAGA |  |
|  |  | GAAGCCTCAGCGCTATTGAG |  |
| ZO-1 |  | GACTCCAGACAACATCCCGAA |  |
|  |  | AACGCTGGAAATAACCTCGTTC |  |
| Claudin 8 |  | CGCTGGAGGAGCACTGTTCTGTTG |  |
|  |  | CGGCGTGGAAACTCCGTTGA |  |
| IL-6 |  | TACCACTTCACAAGTCGGAGGC |  |
|  |  | CTGCAAGTGCATCATCGTTGTTC |  |
|  |  |  |  |
